# Short- and Branched-Chain Fatty Acids as Fecal Markers for Microbiota Activity in Vegans and Omnivores

**DOI:** 10.3390/nu13061808

**Published:** 2021-05-26

**Authors:** Iris Trefflich, Stefan Dietrich, Annett Braune, Klaus Abraham, Cornelia Weikert

**Affiliations:** 1 Department of Food Safety, German Federal Institute for Risk Assessment, 10589 Berlin, Germany; stefan.dietrich@bfr.bund.de (S.D.); klaus.abraham@bfr.bund.de (K.A.); cornelia.weikert@bfr.bund.de (C.W.); 2 Research Group Intestinal Microbiology, Department of Molecular Toxicology, German Institute of Human Nutrition Potsdam-Rehbruecke, 14558 Nuthetal, Germany; braune@dife.de

**Keywords:** vegan diet, short-chain fatty acids, branched-chain fatty acids, fecal pH, intestinal microbiota

## Abstract

A vegan diet could impact microbiota composition and bacterial metabolites like short-chain (SCFA) and branched-chain fatty acids (BCFA). The aim of this study was to compare the concentrations of SCFA, BCFA, ammonia, and fecal pH between vegans and omnivores. In this cross-sectional study (vegans *n* = 36; omnivores *n* = 36), microbiota composition, fecal SCFA, BCFA, and ammonia concentrations and pH were analyzed in complete stool samples. A random forest regression (RFR) was used to identify bacteria predicting SCFA/BCFA concentrations in vegans and omnivores. No significant differences in SCFA and BCFA concentrations were observed between vegans and omnivores. Fecal pH (*p* = 0.005) and ammonia concentration (*p* = 0.01) were significantly lower in vegans than in omnivores, while fiber intake was higher (*p* < 0.0001). Shannon diversity was higher in omnivores compared to vegans on species level (*p* = 0.04) only. In vegans, a cluster of *Faecalibacterium prausnitzii*, *Prevotella copri*, *Dialister* spp., and *Eubacterium* spp. was predictive for SCFA and BCFA concentrations. In omnivores, *Bacteroides* spp., *Clostridium* spp., *Ruminococcus* spp., and *Prevotella copri* were predictive. Though SCFA and BCFA did not differ between vegans and omnivores, the results of the RFR suggest that bacterial functionality may be adapted to varying nutrient availability in these diets.

## 1. Introduction

The community of all living microorganisms across the human body is termed as the “microbiota”, with most microorganisms living in the gut system [1]. The intestinal microbiota, an ecosystem itself, consists mostly of bacteria alongside viruses, fungi, or protozoans with a commensal relationship with the host [2].

Analyses of stool samples of healthy subjects revealed a gene catalogue of approximately 3 million different microbial genes and more than 1000 different bacterial species [3]. The gut microbiota is characterized by a high diversity, thereby Bacteroidetes, Firmicutes, Proteobacteria, and Verrucomicrobia present the most abundant phyla in humans [4].

The majority of gut bacteria have a commensal relationship with the host. The gut microbiota is involved in nutrient metabolism; it is assumed to have protective functions, such as displacement of pathogens, and acts as an intestinal barrier [2]. On the other hand, the microbiota is also implicated in the onset of several diseases, such as inflammatory bowel diseases (IBD) [5] or metabolic disorders [6]. The metabolic activities of the microbiota are complex and have been presented recently in an interspecies interaction network with over 4000 degradation and transport reactions between hundreds of involved species and metabolites [7].

Moreover, gut bacteria produce metabolites, which are formed from dietary substrates and may affect host health [8]. The major products of non-digestible dietary fiber fermentation by bacteria are the short-chain fatty acids (SCFA) acetate, butyrate, propionate, and valerate [9,10,11], of which acetate is the most abundant [10]. Bacterial species such as *Roseburia* spp., *Faecalibacterium prausnitzii*, *Ruminococcus bromii*, or *Prevotella* spp. are known SCFA producers, and their intestinal abundance is positively associated with fiber intake [12,13].

Concentrations of SCFA increase from the ileum to caecum and distal colon [10]. Butyrate is the main energy source for colonocytes and, thus, rapidly absorbed in the colon [14]. A major percentage of SCFA formed by bacteria is absorbed by colonocytes and only a minor part (5%) is excreted with feces [10]. SCFA also act as signaling molecules [14], for example, butyrate and propionate are linked to gluconeogenesis and energy homeostasis [15]. Due to their anti-inflammatory and antiproliferative properties, SCFA may also be associated with a lower risk of IBD or colon cancer [14,16].

To a lesser extent, bacteria produce short branched-chain fatty acids (BCFA), such as isobutyrate and isovalerate, by protein fermentation. Thus, BCFA concentrations are associated with dietary protein intake. Nitrogenous metabolites of protein degradation and amino acid fermentation, such as amines, phenols, or ammonia, are considered to have toxic effects on colonocytes and to induce inflammation [17,18].

Bacterial growth and activity depend on the acidity of gut contents, and intestinal pH may have an impact on bacterial competition [2,11]. A lower pH may promote the growth of SCFA-producing bacteria, and high SCFA concentrations may lower intestinal pH at the same time [13]. Due to the beneficial health effects of SCFA and the strong interplay between pH and SCFA concentrations, a lower fecal pH could indicate improved gut health.

So far, only very few studies investigated SCFA concentrations in stool of participants following a vegan diet containing more vegetables and, thus, fiber compared to omnivorous diets [19,20,21,22]. However, these studies do not provide suitable information about SCFA concentrations in an adult vegan population and have rather a small study size. One study was conducted in children of a rural Africa population [21]; another described changes in SCFA concentration after a short-term intervention [19]; and the further study investigated SCFA concentrations in vegans, vegetarians, and omnivores according to an adherence to a Mediterranean diet [20].

The aim of the present cross-sectional study “Risks and Benefits of a Vegan Diet” (RBVD) was to compare fecal SCFA and BCFA concentrations in vegans and omnivores, and, in addition, fecal pH and ammonia concentrations. To the best of our knowledge, these markers have not been investigated at the same time in a study with healthy vegans compared to omnivores. In addition, the gut microbiota composition of vegans and omnivores was investigated according to observed SCFA and BCFA concentrations.

## 2. Materials and Methods

### 2.1. Study Design

For our cross-sectional study, “Risks and Benefits of Vegan Diet” (RBVD), 36 vegans and 36 omnivorous participants were recruited. The detailed study design has been described previously [23]. In short, participants aged 30–60 years with a body mass index (BMI) < 30 kg/m^2^, and following their diet for at least one year, were included. Vegans were defined as individuals consuming no animal products, and omnivores were defined as individuals consuming at least three portions of meat or at least two portions of meat and two portions of processed meat per week. Participants with previous diseases and pregnant women were not included. The nutrient intake was assessed by 3-day weighed food protocols [24]. Stool, fasting blood, and 24-h urine samples were collected. The study was conducted at the Federal Institute for Risk Assessment, Berlin, Germany. The Ethic Commission of the Charité-Universitätsmedizin Berlin, Germany, approved the study (No. EA4/121/16).

### 2.2. Collection and Processing of Fecal Samples

The participants received a collection device (Fecotainer^®^, AT Medical BV, Enschede, Netherlands) to collect a complete stool sample at home in the morning of the second examination day. The processing of stool samples has been described previously [24,25]. Briefly, the complete stool samples were homogenized for 15 min (Laboratory Blender Stomacher 400, Seward Ltd., West Sussex, UK) and aliquoted for further analysis. Time of defecation, arrival at the study site, and time point of freezing were documented. The samples were stored at −80 °C. The pH value was determined using a pH electrode in the remaining sample (Knick Portamess^®^, Knick GmbH, Berlin, Germany).

### 2.3. Analysis of Fecal Markers

Concentrations of SCFA and BCFA were measured by gas chromatography at the German Institute of Human Nutrition Potsdam-Rehbruecke (DIfE), Nuthetal, Germany. The stool sample aliquot was weighed, diluted with 350 μL of water, and centrifuged (14,000× *g*, 5 min). The supernatant (100 μL) was mixed with 20 μL of 8.6 mM ethyl butyrate (internal standard), 280 μL of 0.36 M perchloric acid, and 270 μL of 1 M sodium hydroxide. After freeze-drying overnight, the residue was dissolved in 100 μL of 5 M formic acid and 400 μL of acetone and centrifuged (14,000× *g*, 5 min). An aliquot (1 µL) of the organic phase was used for the quantification of SCFA and BCFA by a gas chromatograph (HP 5890 series II; Agilent, Waldbronn, Germany) equipped with a HP-20 M column and a flame ionization detector (FID), as described previously [26].

Ammonia levels were determined at DIfE in fecal supernatants following ultrafiltration (Vivacon 500 centrifugal units, Sartorius, Göttingen, Germany) by using a colorimetric assay (Ammonia Colorimetric Assay Kit II, BioVision, Milpitas, CA, USA). Amounts of all compounds refer to fecal wet weight.

### 2.4. Microbiota Analysis and Analysis of Taxa Abundances and Diversity Analysis

Partial 16S ribosomal RNA (rRNA) gene sequencing was performed by CeMeT GmbH (Tübingen, Germany) as described previously [25]. Fecal DNA was isolated with the Stratec InviMag Stool DNA Kit (Invitek, Berlin, Germany) and then treated with Zymo OneStep PCR Inhibitor Removal Kit (Zymoresearch, Irvine, CA, USA). The V3 and V4 regions of the 16S rRNA gene were sequenced using Illumina MiSeq platform (Illumina Inc., San Diego, CA, USA). Reads were merged by using the open-source tool FLASH (Fast Length Adjustment of SHort reads) and subsequently filtered [27]. A sequence library was built, and data were matched with the NCBI Bacterial 16S rRNA database. Taxonomic classification was conducted with the software MALT (Megan Alignment Tool) using the “Lowest Common Ancestor“ algorithms [28].

Within-in sample diversity in vegans and omnivores was described by alpha diversity using Shannon and Simpson diversity indices [29]. The beta-diversity indices, Bray–Curtis dissimilarity, and Jaccard distance [30] were used to describe sample diversity between vegan and omnivorous participants. For this purpose, distance matrices of bacterial species based on Bray–Curtis dissimilarity and Jaccard distance were used to form non-metric multidimensional scaling (NMDS) plots. Differences of abundances of taxa between vegans and omnivores were presented at phylum, genus, and species levels.

### 2.5. Statistics

Characteristics of the study population such as age, sex, educational level and lifestyle items, intake of nutrients, microbiota data, and fecal biomarkers were presented as means and standard deviation (STD) or as median and interquartile range (IQR) for continuous variables. Categorical variables were presented as percentages. For categorical variables, Chi2 or Fishers Exact test was used; for continuous variables, Mann–Whitney U or T-test (for normally distributed variables) was applied. Correlation between variables was calculated using Spearman correlation.

### 2.6. Random Forest Regression Analysis

Random forest regression (RFR) was used to identify bacterial species predictive for SCFA and BCFA concentrations in vegans and omnivores. The outcome of SCFA and BCFA concentrations was predicted based on absolute abundance of bacterial species adjusted for the following potential confounder: age, BMI, sex, physical activity, fecal pH, and dietary intake of fiber, protein, carbohydrates, and fat. Only species were included in the RFR that were (1) present in at least 50% of participants with vegan or omnivorous diet and (2) with an absolute abundance of 100 reads in at least one participant. In order to measure reliable prediction values, a cross-validation (CV) approach with 100 runs was applied. For each outcome, 100 RFR were computed with 2000 trees. For each RFR, randomly 75% of data was used as training and 25% as a test set. To evaluate the predictive power of bacterial species abundance on SCFA and BCFA concentrations, three models for each SCFA and BCFA were calculated: (1) a baseline model with only potential confounders, (2) a full model with potential confounder and all included species (*n* = 50), and (3) a final model with potential confounder and predictive selected species. The full model was used to identify predictive species based on percentage increase in mean square error (%IncMSE). A threshold of >2 %IncMSE across 100 CV runs was used to select predictive species. Higher values for %IncMSE indicate more predictive species regarding the respective B/SCFA. A heatmap was generated to visualize the %IncMSE values of the species for each B/SCFA from the full model. To determine the predictive quality of the models in terms of predicting the SCFA and BCFA concentrations by the species in addition to the confounder, the mean of the explained variance (R^2^) and the mean of the root mean square error (RMSE) across 100 CV runs were calculated.

(1) $$\mathrm{Formula}\;\mathrm{of}\;\mathrm{RMSE}:\;\mathrm{RMSE}\;=\;\sqrt{\frac{\sum_{i=1}^{n}(x_{i}-y_{i}{)}^{2}}{n}}$$

(2) $${\mathrm{Formula}\;\mathrm{of}\;R}^{2}:R^{2}\;=\;1-\frac{\sqrt{\sum_{i=1}^{n}(y_{i}-x_{i}{)}^{2}}}{\sqrt{\sum_{i=1}^{n}(y_{i}-\bar{y}{)}^{2}}}$$

In the formulas, *x* represents the predicted SCFA or BCFA concentrations and *y* represents the actual SCFA or BCFA concentrations, respectively.

The RMSE is measured in the same unit as the SCFA, which complicates a comparative interpretation when using multiple outcomes. Therefore, to interpret the RMSE in terms of percentage of the mean, the RMSE was divided by the mean of the respective SCFA and BCFA. Statistical analyses were conducted with SAS, v9.4 (SAS Institute, Cary, NC, USA). and R (v3.6.3) using the packages *vegan*, *randomForest*, and *pheatmap*.

## 3. Results

### 3.1. Description of Study Population Including Macro Nutrient Intake

The main characteristics and macronutrient intake of the study population are given in Table 1 and have been published previously [23]. The study population was sex- and age-matched. Lifestyle factors, such as physical activity or smoking, BMI, and educational level did not differ significantly between vegans and omnivores, which may be attributed to the small study size. Vegan participants followed their diet on average 4.8 years (IQR 3.1–8.7). Dietary fiber intake (Table 1) was higher in vegans than in omnivores (*p* ≤ 0.0001), whereas protein (*p* = 0.02) and fat (*p* = 0.004) intake was lower in vegans compared to omnivores. Total energy intake did not differ significantly between the two groups (*p* = 0.32).

### 3.2. Short- and Branched-Chain Fatty Acids

**Figure 1:**
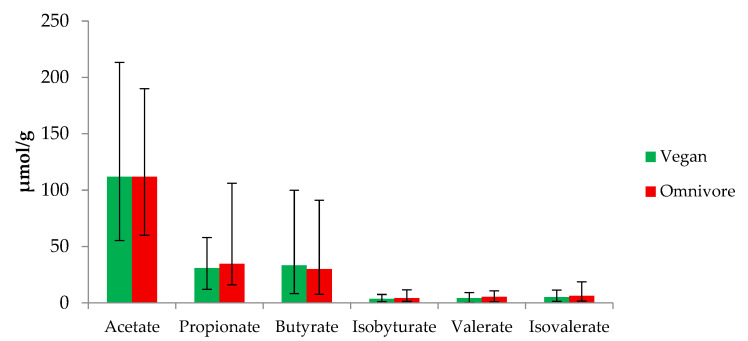
Concentrations of short and branched chain fatty acids in stool of RBVD (Risks and Benefits of Vegan Diet) population (µmol/g). Data are presented as median and interquartile range (Q1–Q3).

Concentrations of SCFA and BCFA did not differ significantly between vegans and omnivores (Figure 1). Nevertheless, some trends were observed between the two groups of diet. Propionate concentrations tended to be higher in omnivores (34.70 µmol/g, IQR 26.05–41.65) than in vegans (30.85 µmol/g, IQR 23.55–37.15) (*p* = 0.15). Concentrations of butyrate tended to be higher in vegans (33.25 µmol/g, IQR 25.85–42.80) compared to omnivores (29.95 µmol/g, IQR 26.05–41.65) (*p* = 0.33). In vegans, concentrations of isovalerate (5.20 µmol/g, IQR 3.20–7.75), valerate (4.15 µmol/g, IQR 3.25–5.40), and isobutyrate (3.65 µmol/g, IQR 2.25–4.85) tended to be lower than those in omnivores (isovalerate, 6.15 µmol/g, IQR 4.4–8.05; valerate, 5.40 µmol/g IQR 3.45–7.05; isobutyrate, 4.20 µmol/g, IQR 3.0–5.1).

### 3.3. Fecal pH and Ammonia

Median stool weight (*p* = 0.79) and daily stool frequency (*p* = 0.34) did not differ between vegans and omnivores. The fecal pH value of omnivores (6.73 ± 0.45) was higher than that of vegans (6.41 ± 0.48) (*p* = 0.005). Ammonia levels were lower in vegans (25.05 µmol/g, IQR 17.02–35.09) than in omnivores (32.13 µmol/g, IQR 27.21–38.58) (*p* = 0.01) (Table 2).

### 3.4. Fecal Microbiota Composition

**Figure 2:**
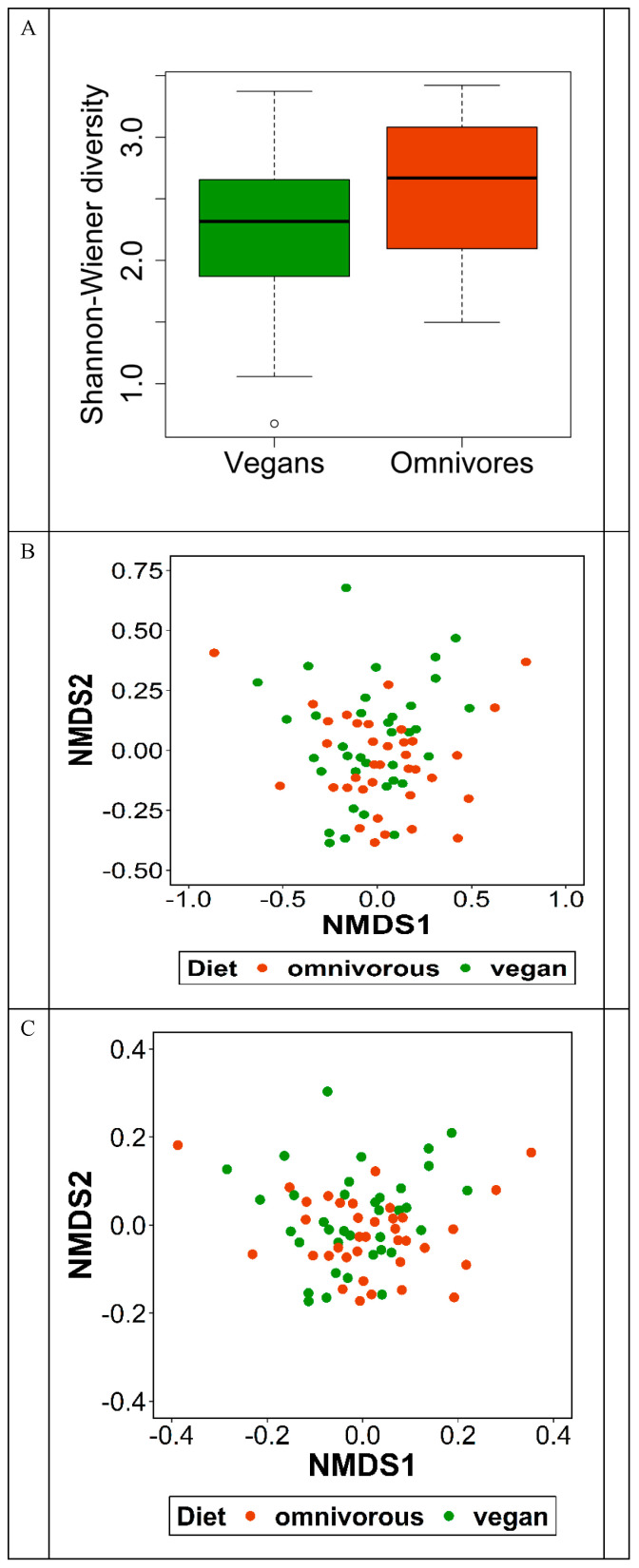
Alpha and beta diversity of gut microbiota in RBVD. Alpha diversity presented as Shannon Index (**A**). Beta diversity in vegans and omnivores presented as (**B**) Bray–Curtis and (**C**) Jaccard distance.

The bacterial 16S rRNA sequence analysis revealed in total 27 phyla, 48 classes, 226 families, 687 genera, and 1195 species in the study population (Table 3). The mean number of detected species was 174.2 (±35.4) in vegans and 172.0 (±39.4) in omnivores (Supplementary Table S1). At each taxonomic level, there was no difference in alpha diversity, described as Simpson index, between vegans and omnivores. Alpha diversity, described as Shannon diversity, was higher in omnivores than in vegans on species level (*p* = 0.04) (Figure 2). At all other taxonomic levels, no difference in alpha diversity was observed (Supplementary Table S1). NMDS plots based on the beta-similarity indices, Bray–Curtis dissimilarity and Jaccard distance, indicate no differences in the abundances of species between vegans and omnivores (Figure 2).

**Figure 3:**
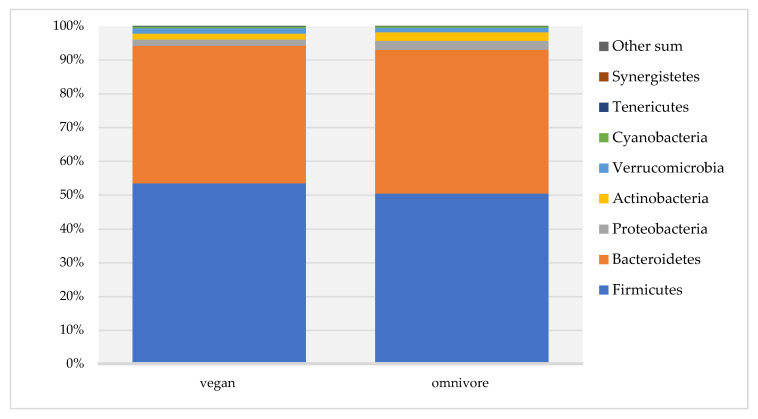
Gut microbiota composition at phylum level in RBVD.

Bacteriodetes, Firmicutes, Proteobacteria, Actinobacteria, Cyanobacteria, and Verrucomicrobia were the six most abundant phyla in both dietary groups, and the number of reads did not differ between vegans and omnivores. Only the abundance of members of the phylum Tenericutes was significantly higher in vegans (median number of reads 13.5, IQR 0–56) compared to omnivores (number of reads 1.0, IQR 0–12.8) (*p* = 0.02) (Supplementary Table S2, Figure 3).

**Figure 4:**
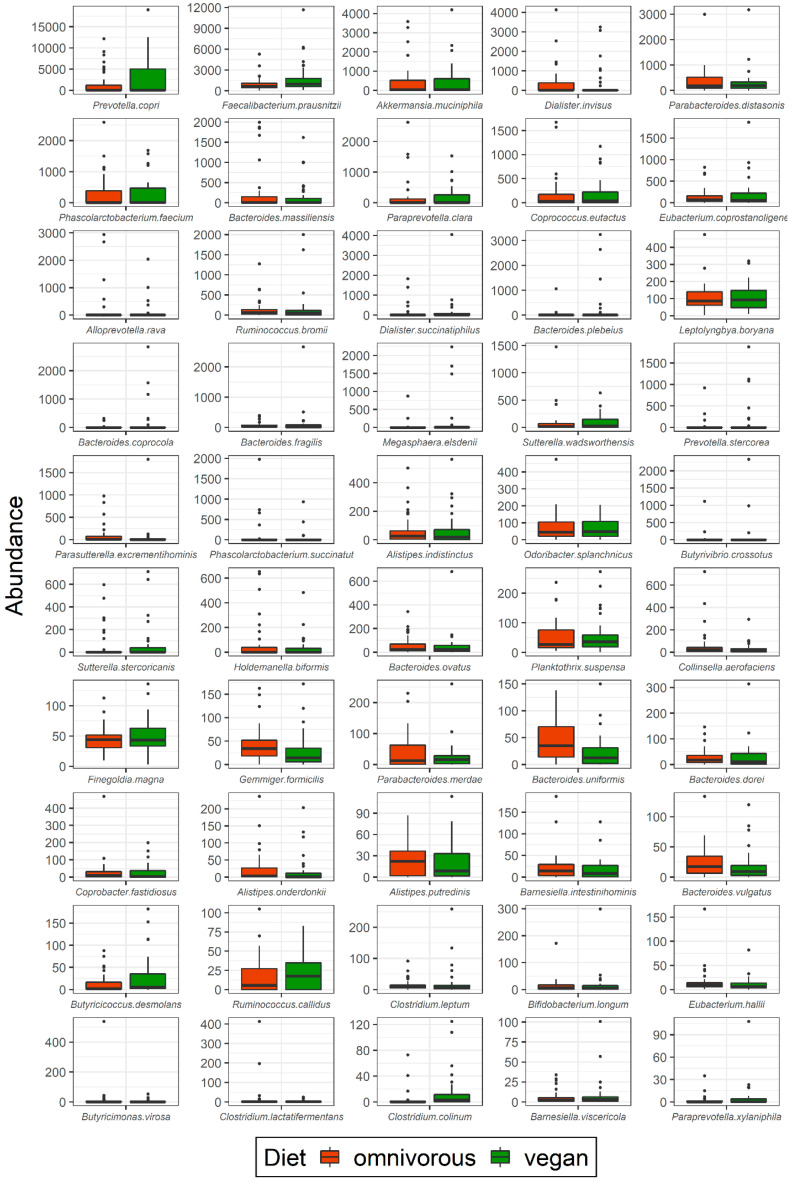
Gut microbiota composition at species level in vegans and omnivores. Abundances of bacteria at species level in vegans and omnivores are presented as number of reads using 16S rRNA sequencing. Significant differences were observed for *Butyricicoccus desmolans* (*p* = 0.049)*, Clostridium colinum* (*p* = 0.004)*, Dialister succinatiphilus* (*p* = 0.02), *Bacteroides uniformis* (*p* = 0.004), *Bacteroides vulgatus* (*p* = 0.07)*, Parasutterella excrementihominis* (*p* = 0.04), and *Dialister invisus* (*p* = 0.04).

On species level, the abundance of *Butyricicoccus desmolans* (*p* = 0.049)*, Clostridium colinum* (*p* = 0.004), and *Dialister succinatiphilus* (*p* = 0.02) was significantly higher in vegans than in omnivores (Supplementary Table S3). In omnivores, the absolute abundances of *Bacteroides uniformis* (*p* = 0.004), *Bacteroides vulgatus* (*p* = 0.07)*, Parasutterella excrementihominis* (*p* = 0.04), and *Dialister invisus* (*p* = 0.04) were higher compared to vegans. *Faecalibacterium prausnitzii* was the most abundant species in all participants; though the number of reads was higher in vegans (median 968.5, IQR 580.8–1764.2) than in omnivores (median 637.0, IQR 440.2–1105.0), the difference remained non-significant (*p* = 0.077) (Supplementary Table S3). Absolute abundances of species in the study population are presented in Figure 4. Absolute abundances at genus level are presented in Supplementary Table S4.

### 3.5. Identification of Bacterial Species Predictive for SCFA and BCFA Concentrations

**Figure 5:**
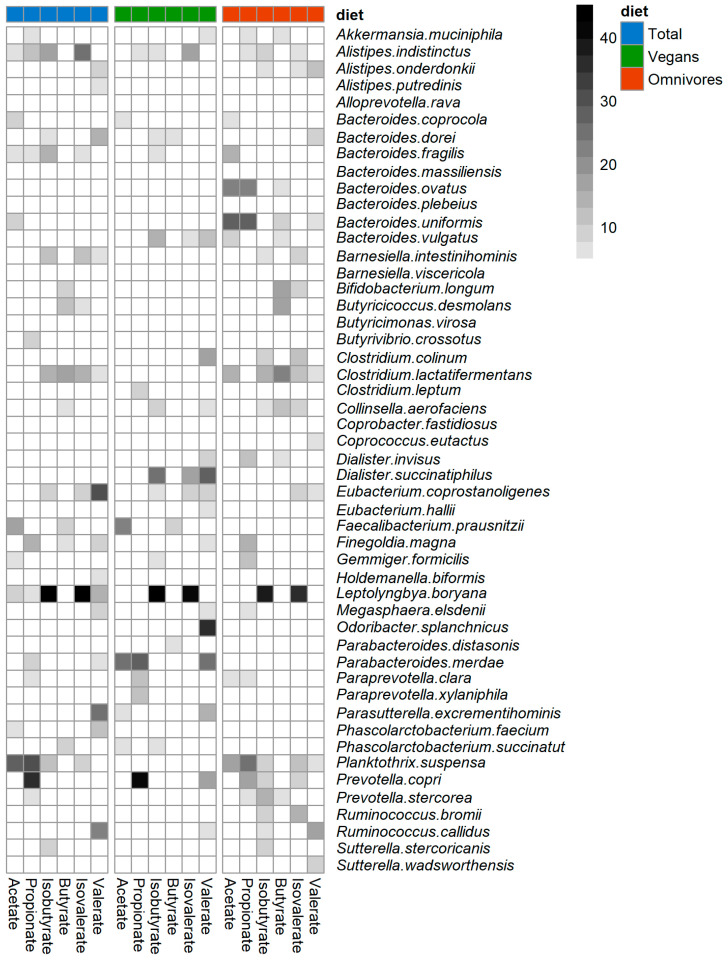
Predictive bacteria for selection model. Grid cells shows the mean square error (MSE%) values of bacteria with an MSE% value >2 from the selection model. The selection model was computed using S/BCFA as outcomes and the most abundant bacteria (50% of participants and >100 reads) as exposure variables adjusted for age, sex, BMI, physical activity, smoking status, stool pH, protein, fat, carbohydrates, fiber, and alcohol intake. The darker the MSE% value, the more important a bacterial species is for the prediction of the respective S/BCFA.

To identify bacterial species predictive for the SCFA and BCFA concentrations, RFR models were computed for the total study population, vegans, and omnivores. In omnivores, a pattern of species belonging to *Bacteroides*, *Clostridium*, and *Prevotella* was identified to be predictive for SCFA and BCFA (Figure 5), whereas in vegans, partly other species were identified. These differences are not visible when looking at the findings for the total sample only. Looking at acetate only (Figure 5), the following species were highly predictive in omnivores: *Bacteroides fragilis*, *B. ovatus, B. uniformis*, *B. vulgatis*, *Clostridium lactatifermentans*, and *Planktothrix suspense*, whereas in vegans only *F. prausnitzii* and *Parabacteroides merdae* were highly predictive. Such differences between vegans and omnivores for the selection of the predictive bacteria were also evident for the other SCFA and BCFA (Figure 5). Moreover, it is noticeable that only *F. prausnitzii* was predictive for butyrate and acetate in vegans. *D. succinatiphilus* was predictive for BCFA in vegans. *Prevotella copri* was highly predictive for propionate and valerate in vegans, whereas in omnivores, *P. copri* was predictive for propionate, isobutyrate, and isovalerate.

When looking at the prediction performance of the identified bacteria (Table 4), it becomes evident that the explained variances (R^2^) of the final models (confounder + predictive bacteria) were higher than those of the baseline (only confounder) and the full models (confounder + all 50 bacteria). This indicates that the selected species improves the prediction of the SCFA and BCFA when added to covariate data. Note that negative R^2^ values or values close to zero are a sign of overfitting and indicate non-predictive RFR models. For example, in vegans, the explained variance of acetate was lower in the full model (R^2^ = −0.05) compared to the baseline model (R^2^ = 0.10) suggesting overfitting due to many non-informative bacterial variables. However, the prediction performance of the full model (R^2^ = 0.21) was improved compared to the baseline model, suggesting that in addition to the confounder, the selected bacteria, *F. prausnitzii* and *P. merdae*, contributed to the prediction of acetate in vegans. The final models of vegans (R^2^ = 0.30) showed lower explained variance than those of omnivores (R^2^ = 0.45) for propionate (R^2^ = 0.30 and R^2^ = 0.45) and acetate (R^2^ = 0.21 and R^2^ = 0.36). By contrast, the final models of vegans showed higher explained variance than the ones of omnivores for valerate (R^2^ = 0.38 and R^2^ = 0.24) and iso-butyrate (R^2^ = 0.28 and R^2^ = 0.16). Only small differences for the explained variance were found for butyrate (R^2^ = 0.27 and R^2^ = 0.26) and isovalerate (R^2^ = 0.21 and R^2^ = 0.18). However, the RMSE values indicate a moderate degree of uncertainty in the prediction. Lower RMSE values indicate RFR models with lower error rate compared to RFR models with higher RMSE values. In terms of RMSE, the best predictive quality was achieved by the final models for acetate in vegans (23.5%) and omnivores (21.3%).

### 3.6. Correlations of SCFA and BCFA Concentrations, pH, and Ammonia Levels and with Dietary Nutrients

**Figure 6:**
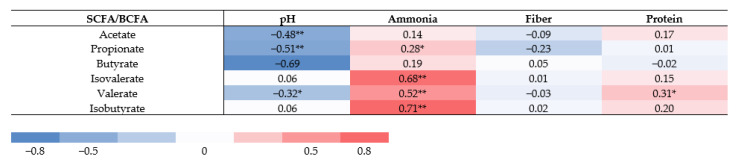
Spearman correlation between SCFA/BCFA concentrations, fecal metabolic markers, and dietary variables. Correlation presented with Spearman correlation coefficient. Significant differences marked with asterisks (** *p* = < 0.0001, * *p* ≤ 0.05).

Correlations between S/BCFA, pH, and ammonia, and between macronutrients were assessed (Figure 6). Fecal pH correlated inversely with concentrations of acetate (*r* = −0.48), propionate (*r* = −0.51), and valerate (*r* = −0.32), but not with isobutyrate (*r* = 0.06) and isovalerate (*r* = 0.06). There was no significant correlation between fiber intake and all fatty acids. Only valerate concentrations showed a correlation with protein intake (*r* = 0.31, *p* = 0.009). Ammonia levels correlated positively with propionate (*r* = 0.28, *p* = 0.05), valerate (*r* = 0.52, *p* < 0.0001) and BCFA concentrations (isovalerate, *r* = 0.68; isobutyrate, *r* = 0.71) (*p* = < 0.0001).

## 4. Discussion

To our knowledge, this is the first study presenting fecal concentrations of SCFA, BCFA, and ammonia and pH levels at the same time in vegans compared to omnivores. In this cross-sectional study, fecal concentrations of these markers were investigated in a healthy and Western vegan population. Moreover, the intestinal microbiota composition of vegans was compared with that of omnivores.

The high intakes of vegetables and fruits and, thereby, fiber are suggested as major factors contributing to health benefits of a vegan diet [31]. Dietary fiber may considerably impact gut microbial metabolism [13], and the fermentation of non-digestible fiber results in the production of SCFA by gut bacteria [9,10,11]. Bacterial species such as *Roseburia* spp., *Faecalibacterium prausnitzii*, *Ruminococcus bromii*, and Prevotella spp. ferment fiber into SCFA. Higher abundance of Prevotella species has been reported in cross-sectional studies with vegan [20,32,33] and vegetarian [32,34] populations, which might be attributed to higher fiber intake with these types of diet. In an intervention study, SCFA concentrations correlated positively with the abundances of *Roseburia*, *Faecalibacterium* and *Bifidobacterium* [19]: after a 6-day intervention with a plant-based diet, the subjects showed significantly higher acetate and butyrate levels compared to subjects from the animal-based dietary intervention group [19]. In a cross-sectional study with vegan, vegetarian, and omnivorous participants who followed their diet for at least one year, SCFA concentrations were higher in the vegan group compared to omnivores. Nevertheless, the authors also observed a positive correlation between SCFA concentrations and persons with a higher adherence to Mediterranean diet, rich in fruit, vegetables, and legumes regardless of the type of diet [20]. Higher SCFA concentrations were also observed in a cross-sectional study in African children with a predominant plant-based diet, rich in fiber and low in animal fat compared to a juvenile population with a Western diet [21]. Though different SCFA concentrations were observed in populations following a vegan diet rather long-term [20,21], this did not apply for our study, where participants consumed nearly five years a vegan diet.

Butyrate concentrations tended to be higher in vegans than in omnivores of our study, but in contrast, and despite higher fiber intake, our study could not demonstrate significant differences in fecal SCFA concentrations between the two groups of diet. Our observations are in line with another cross-sectional study in a healthy Western vegan population, who followed their diet for at least six months [22]. The authors suggested that the microbiota structure of the Western population is “restrictive” and SCFA production does not increase linearly with fiber availability, as it occurs in an agrarian society with a “permissive” microbiota structure [22]. In animal models, it has been shown that feeding mice over generations with a diet low in fiber resulted in a loss of microbiota diversity and taxa [35]. Moreover, after switching back to high-fiber diet, microbiota composition did not restore in the following generations, thus confirming the hypotheses of shifts in microbiota activity over generations due to environmental factors including diet. Taken together, these assumptions may explain the similar SCFA concentrations in vegans and omnivores despite different fiber intake observed in our study.

BCFA result from bacterial degradation of proteins and fermentation of mainly branched-chain amino acids, valine, leucine, and isoleucine [17,18]. BCFA are discussed as markers of protein fermentation, and diets high in protein are associated with higher BCFA levels [36]. Although protein intake was higher in omnivores than in vegans, we observed only a trend of lower BCFA concentrations in vegans compared to omnivores, but no statistical significant difference in BCFA concentrations. Moreover, BCFA concentrations did not correlate with protein intake of our study population. In an in vitro study, lower BCFA formation from proteins was observed following an anaerobic incubation with fecal suspensions of vegetarians compared to incubation with fecal slurries of omnivorous subjects [37]. Bacteria from vegetarian donors grew faster on soy protein as substrate, while in omnivorous samples, meat protein and casein were the preferred growth substrates [37].

We observed significantly lower fecal ammonia concentrations in vegans compared to omnivores. Ammonia, as a metabolite of bacterial protein degradation and amino acid fermentation, is discussed to be toxic to colonocytes and to cause inflammation in mammals [17,18]. Although we could not observe a correlation between fecal ammonia concentrations and protein intake, BCFA levels correlated strongly with ammonia concentrations. This is in line with the results of another in vitro study, using fecal samples of healthy donors for an anaerobic fermentation [38]. In this study, dietary behavior of the previous year was assessed by questionnaire. The authors observed an association between BCFA and ammonia formation, and, moreover, BCFA and ammonia concentrations were positively associated with the intake of processed meat and dairy products [38].

Intestinal pH plays an important role for bacterial growth and activity. In the distal colon, pH is higher than in the proximal colon, where SCFA production may lower pH [39]. On the other side, dietary fiber impacts intestinal transit time, and a decreased transit time leads to elevated SCFA concentrations and lower fecal pH [13]. As shown in an in-vitro study, *Bacteroides* preferred alkaline pH for growth and was inhibited at a pH lower than 6.5 [40]. In contrast, *F. prausnitzii* preferred lower pH, and growth rates were higher at pH 5.5 compared to pH 6.5 under anaerobic conditions [40]. In line with these findings, we observed an inverse correlation of pH and SCFA concentrations in our RBVD study. Moreover, several *Bacteroides* species were more abundant in omnivores than in vegans in our study, which might correspond to higher fecal pH in omnivores. In line with this as well, *F. prausnitzii* was more abundant in vegans than in omnivores, which might be related to the lower fecal pH in the vegan group.

While the microbiota composition and fecal SCFA and BCFA concentrations showed only modest differences between vegans and omnivores, the Random Forest Regression (RFR) analysis revealed different clusters of species predicting SCFA and BCFA concentrations in vegans and omnivores. Several species have been identified, which are involved in SCFA formation [9,11], and correlations between those bacteria and SCFA were also observed in an intervention study after a six-day change from an animal-based diet to a plant-based diet [19]. In the vegans of our study, *Dialister succinatiphilus, F. prausnitzii*, and *P. copri* were predictive for SCFA concentrations in stool. *F. prausnitzii* is one of the major butyrate producers in the human gut [11]. Two recent reviews summarized associations of diet and *F.* *prausnitzii* counts [41] and this species’ beneficial effects in IBD [42]. Interestingly, in our study, *F. prausnitzii* was predictive for acetate and butyrate concentrations in vegans only, but not for any SCFA or BCFA in omnivores. Other butyrate-producing bacteria [9,14], such as *Roseburia*, *Eubacterium*, or *Fusobacterium* species, were not identified by RFR, which might be due to low abundances of these taxa in our study population. A cluster of *B. uniformis, B. ovatus, Bifibacterium longum*, and *Clostridium* spp. were predictive for SCFA and BCFA concentrations in omnivores, but not in vegans. Moreover, *B. uniformis* and *B. ovatus* were significantly more abundant in omnivores than in vegans in our study. *Bacteroides* members are associated with propionate formation [9], and in our study, species belonging to *Bacteroides* were predictive for propionate concentrations. In contrast, *D. succinatiphilus* numbers were significantly higher in vegans than in omnivores and predicted BCFA levels only in vegans. This is surprising because *D. succinatiphilus* has been associated with carbohydrate degradation and production of propionate [43], rather than with protein fermentation resulting in BCFA production.

It has become evident that the intestinal microbiota composition of human subjects is heterogeneous and driven by high individuality [44]. Although several studies have been conducted to investigate the impact of vegan or plant-based diets on gut microbiota, so far no consistent and universally applicable results could be obtained [25]. Against this background, our findings regarding diet-dependent fecal microbiota composition need to be interpreted with caution.

Our study had some limitations. The study size was relatively small with 72 participants, which may have led to a low statistical power. Due to the cross-sectional study design, which included collection of only one single stool sample, time-dependent changes in SCFA concentrations or microbiota composition could not be monitored. The majority of SCFA and BCFA are absorbed by colonocytes, thus SCFA concentrations in feces do not completely reflect the synthesis rate [17]. Colonocyte metabolism may also play a role in shaping intestinal microbiota and may be considered in the context of fecal SCFA concentrations. Colonocytes oxidize fatty acids including butyrate, which leads to an increased epithelial oxygen consumption [45]. The resulting hypoxia promotes the abundance of obligate anaerobic bacteria, which convert fiber into SCFA. Colonocytes can change their metabolism to anaerobic glycolysis, thereby shifting the microbiota to more facultative anaerobic bacteria [45] that belong to the phylum Proteobacteria and are implicated in dysbiosis of the microbiota. In our study, we observed only a low abundance of Proteobacteria, whereas anaerobic Firmicutes and Bacteroidetes were the most abundant phyla. The absorption rate of SCFA by the colonocytes may adapt in vegans and omnivores, which would explain the similar SCFA amounts excreted with stool. Further research is required to unravel the underlying mechanism and to draw conclusions.

Nevertheless, the concentrations and ratios of SCFA and BCFA observed in RBVD were similar to findings in healthy subjects reported in previous studies [17,46]. Moreover, the fast and standardized processing of complete stool samples and keeping at −80 °C before laboratory analyses, as done in our study, should have prevented major metabolic changes to occur in the collected stool samples [47]. In this study, microbiota composition was analyzed by using 16S rRNA sequencing, which gives information about bacterial abundances, but not about bacterial functions. However, microbiota composition was examined in parallel with microbial metabolites and pH which may reflect the microbiota as a complex community and network [7].

In our study, diet was assessed with a 3-day weighed food protocol and using the German Nutrient Data Base. This database summarizes different types of non-digestible carbohydrates by the term “fiber” and does not allow detailed information on their specific characteristics and the fermentation rate by the gut microbiota [48].

Our study investigated the complex interplay of gut microbiota, their substrates ingested with diet and derived bacterial metabolites. Despite higher fiber intake in vegans compared to omnivores, SCFA and BCFA concentrations did not differ considerably between the two groups of diet. Yet, our results of the RFR revealed different clusters of species predictable for SCFA and BCFA concentrations in vegans and omnivores. Based on our results of SCFA and BCFA analysis and RFR, we hypothesize that bacterial metabolism might be altered and adapted in vegans and omnivores. Regardless of different nutrient availability, SCFA levels must be maintained as stable to ensure energy supply of colonocytes.

If dietary fiber as a source of SCFA production is limited, bacteria of the gut microbiota switch to amino acids originating from dietary proteins for energy supply [49]. This capability of phylogenetically unrelated species to perform similar functions and to metabolize different substrates to identical metabolites is described as functional redundancy [50]. Using a multi-omics approach, a recent publication revealed varying metabolic pathways in vegans, vegetarians, and omnivores [51], using data from the above-mentioned cross-sectional study by De Filippis [20]. Fecal samples of vegetarians and vegans contained more carbohydrate-metabolizing enzymes than omnivores. Moreover, reconstruction of SCFA-producing pathways revealed that gut bacteria of vegans and vegetarians activated other pathways leading to butyrate and acetate formation than omnivores [51]. These findings could explain that we observed correlations between SCFA and BCFA, and microbial markers like pH and ammonia, but not between fiber or protein intake and SCFA and BCFA. This supports our assumption that bacterial activity and functionality is somehow adapted to dietary habits, as the observed amounts of SCFA and BCFA resulting from microbial fermentations in the gut did not differ between vegans and omnivores.

However, our findings are based on a relatively small number of study participants and, thus, might not be completely transferable to the general population. Moreover, the complexity of the microbiota and their interaction with the host metabolism need to be investigated in-depth by metabolomics studies, taking this interplay of microbiota, metabolites, and diet into account. Long-term studies are also required to unravel the effects of specific dietary types such as vegan diets.

## Figures and Tables

**Table 1:** Characteristics and nutritional intake of RBVD population.

Characteristics	Vegan (*n* = 36)	Omnivore (*n* = 36)	*p*-Value
Male (%)	50	50	
Age (years)	37.5 (32.5–44.0)	38.5 (32.0–46.0)	0.75
Body weight (kg)	70.1 (±13.9)	73.6 (±10.3)	0.24
BMI	22.9 (±3.2)	24.0 (±2.1)	0.08
Duration of vegan diet (years)	4.8 (3.1–8.7)	n.a.	
Education [*n* (%)]			0.60
Lower	0 (0)	1 (2.8)	
Middle	11 (30.6)	11 (30.6)	
High	25 (69.4)	24 (66.7)	
Physical activity (h/week)	2.8 (0.9–3.8)	2.3 (1.2–4.1)	0.69
Smoking behavior [*n* (%)]			0.3
Non-smoker	24 (66.7)	21 (58.3)	
Ex-Smoker	8 (22.2)	6 (16.7)	
Smoker	4 (11.1)	9 (25)	
Nutritional intake			
Total Energy (kcal)	2270 (1800–2762)	2386 (2081–2737)	0.32
Fiber (g/days)	46 (34–56)	24 (19–30)	<0.0001
Proteins (g/days)	72 (55–92)	86.3 (71–107)	0.02
Fat (g/days)	86 (64–111)	104.1 (88–143)	0.004
Carbohydrates (g/days)	259 (212–371)	230.3 (199–291)	0.12

Results presented as means (±STD (standard deviation)), median (Q1–Q3), absolute numbers or percentages (*n* (%)). Statistical tests were done with *t*-test for normal distributed variables, Mann–Whitney U test for not normal distributed variables and Chi^2^ or Fishers Exact for categorical variables. n.a. = not applicable.

**Table 2:** Stool characteristics and fecal biomarkers in RBVD.

Characteristic/Marker	Vegan	Omnivore	*p*-Value
Stool weight (g)	102.6 (40.0–185.9)	97.3 (47.7–157.7)	0.79
Frequency stool (*n* (%))			0.34
<2–3 times/week	0 (0)	0 (0)	
2–3 times/week	2 (5.7)	3 (8.3)	
4–5 times/week	3 (8.3)	8 (22.2)	
daily	23 (63.9)	17 (47.2)	
>daily	8 (22.2)	8 (22.2)	
pH	6.41 (±0.48)	6.73 (±0.45)	0.005
Ammonium (µmol/g)	25.1 (17.0–35.1)	32.1 (27.2–38.6)	0.01

Fecal biomarkers presented as median (Q1–Q3) or mean (±standard deviation), Mann–Whitney U test or *t*-test (for normal distributed variable) was used.

**Table 3:** Shared and unique taxa of gut microbiota in RBVD.

Taxa	(*n*)	Shared in Both Diets *n* (%)	Present in Vegans Only (*n*)	Present in Omnivores Only (*n*)
Phylum	27	23 (85.19)	2	2
Class	48	40 (83.33)	4	4
Family	226	167 (73.89)	22	37
Genus	687	425 (61.86)	128	134
Species	1195	664 (55.56)	272	259

Number of shared and unique taxa at different taxonomic levels in vegans and omnivores of RVBD study. Data are presented as absolute numbers (*n*) and percentages (%).

**Table 4:** Prediction performance of Random Forest regression models for SCFA and BCFA concentrations.

Model	S/BCFA	Vegans		Omnivores		Total Sample	
		RMSE	R^2^	RMSE	R^2^	RMSE	R^2^
Baseline ^a^	Acetate	26.4	0.10	24.7	0.16	24.6	0.20
	Propionate	32.1	0.05	35.2	0.36	31.6	0.30
	Butyrate	44.1	0.21	49.4	0.3	48.9	0.21
	Isobutyrate	45.0	−0.02	46.5	−0.31	47.0	−0.08
	Valerate	46.0	−0.04	40.3	0.13	45.9	0.08
	Isovalerate	48.5	−0.05	49.1	−0.30	50.8	−0.13
Full ^b^	Acetate	29.4	−0.05	23.5	0.17	25.2	0.11
	Propionate	31.1	0.13	37.6	0.32	34.2	0.27
	Butyrate	47.7	0.08	48.3	0.10	50.8	0.17
	Isobutyrate	42.9	0.12	39.0	0.06	43.5	0.06
	Valerate	37.3	0.28	40.1	0.10	42.8	0.17
	Isovalerate	48.2	0.04	41.4	0.05	44.2	0.05
Final ^c^	Acetate	23.5	0.21	21.3	0.36	24.0	0.26
	Propionate	29.5	0.30	32.1	0.45	29.6	0.43
	Butyrate	41.2	0.27	40.2	0.26	45.4	0.30
	Isobutyrate	37.6	0.28	36.5	0.16	40.2	0.21
	Valerate	38.0	0.38	35.5	0.24	40.6	0.30
	Isovalerate	45.4	0.21	37.1	0.18	42.1	0.20

Random forest regression models were computed using cross-validation with 100 runs and S/BCFA as outcomes. ^a^ Baseline model was computed using age, sex, BMI, physical activity, smoking status, stool pH, protein, fat, carbohydrates, fiber, and alcohol intake as exposure variables. ^b^ The full model was computed using all 50 bacteria (50% of participants and >100 reads) as exposure variables adjusted for all variables of baseline model. ^c^ The final model was computed using only selected bacteria with MSE% > 2 as exposure adjusted for all variables of the baseline model. Abbreviations: RMSE, root mean square error; R^2^, explained variance; S/BCFA, short/branch chain fatty acids.

## Data Availability

The datasets generated and/or analyzed during the current RBVD study are not publicly available due to provisions of the data protection regulations.

## Supplementary Materials

The following are available online at https://www.mdpi.com/article/10.3390/nu13061808/s1, Table S1: Within-in sample diversity in vegans and omnivores of RBVD study, Table S2: Abundances of taxa at phylum level in vegans and omnivores, Table S3: Abundances of taxa at species level in vegans and omnivores, Table S4: Abundances of taxa at genus level in vegans and omnivores.
