# Supplementary material for: Short- and Branched-Chain Fatty Acids as Fecal Markers for Microbiota Activity in Vegans and Omnivores
Source: Nutrients. 2021 May 26;13(6):1808. doi: 10.3390/nu13061808 (PMC8230270; doi:10.3390/nu13061808)
Supplement: Supplementary file 1 [file nutrients-13-01808-s001.zip › nutrients-1230781-supplementary.pdf]

Supplementary Table S1. Within-in sample diversity in vegans and omnivores of RBVD study.

Taxonomic Level	Vegans	Omnivores	*p*-Value
**Phylum**			
number of phyla	9.9 (± 1.9)	9.5 (± 1.9)	*0.4*
Pielou's evenness	0.39 (± 0.06)	0.41 (± 0.07)	*0.3*
Shannon diversity	0.89 (± 0.12)	0.90 (± 0.12)	*0.6*
Simpson diversity	0.52 (0.06)	0.52 (± 0.06)	*0.6*
**Class**			
number of classes	20.2 (± 2.7)	19.8 (± 2.6)	*0.5*
Pielou's evenness	0.37 (± 0.5)	0.37 (± 0.5)	*0.6*
Shannon diversity	1.10 (± 0.15)	1.11 (± 0.14)	*0.8*
Simpson diversity	0.57 (± 0.07)	0.57 (± 0.06)	*1*
**Family**			
number of families	66.3 (± 11.3)	67.5 (± 11.4)	*0.1*
Pielou's evenness	0.46 (± 0.07)	0.46 (± 0.05)	*0.9*
Shannon diversity	1.93 (± 0.32)	1.95 (± 0.26)	*0.8*
Simpson diversity	0.77 (± 0.10)	0.77 (± 0.07)	*0.7*
**Genus**			
number of genera	123.2 (± 23.4)	123.2 (± 26.1)	*0.3*
Pielou's evenness	0.45 (± 0.08)	0.45 (± 0.07)	*1*
Shannon diversity	2.18 (± 0.43)	2.18 (± 0.38)	*1*
Simpson diversity	0.75 (± 0.12)	0.76 (± 0.09)	*0.8*
**Species**			
number of species	174.2 (± 35.4)	172 (± 39.4)	*0.8*
Pielou's evenness	0.44 (± 0.12)	0.50 (± 0.10)	*0.03*
Shannon diversity	2.27 (± 0.62)	2.56 (± 0.58)	*0.04*
Simpson diversity	0.74 (± 0.16)	0.81 (± 0.12)	*0.05*

Within-in sample diversity of gut microbiota between vegans and omnivores described by alpha-diversity indices. Data were presented as means (± standard deviation). Significance level was set at $p \leq 0.05$.

Supplementary Table S2. Abundances of taxa at phylum level in vegans and omnivores.

**Phylum**	**Vegans**	**Omnivores**	***p*-Value**	**Vegans (Max)**	**Omnivore (Max)**	**Presence (Vegan)**	**Presence (Omnivore)**
Firmicutes	17357.5 (13550–22231.5)	15603 (12928–18595.5)	0.16	37648	43768	36	36
Bacteroidetes	12127 (9552–16383)	12958 (8857–16517)	0.84	26267	42958	36	36
Proteobacteria	426.5 (204–875.5)	544 (311–997.5)	0.21	4646	3810	36	36
Actinobacteria	347.5 (133–677.5)	266 (143.5–555)	0.73	3643	11041	36	36
Cyanobacteria	153 (96–250.5)	149.5 (96–200)	0.77	406	501	36	36
Verrucomicrobia	32.5 (3–697)	40 (1.5–566)	0.61	4397	3775	35	32
Fusobacteria	2 (1–4)	2 (1–3.5)	0.44	15	28	32	33
Synergistetes	2.5 (0.5–6.5)	2 (1–7)	0.88	102	34	27	28
Tenericutes	13.5 (0–60)	1 (0–13.5)	0.02	253	98	26	21
Euryarchaeota	0 (0–3)	0 (0–0)	0.06	926	89	14	7
Acidobacteria	0 (0–1)	0 (0–1)	0.72	2	3	13	11
Lentisphaerae	0 (0–0)	0 (0–0.5)	0.39	4	13	6	9
Spirochaetes	0 (0–0)	0 (0–0)	0.09	1	1	5	1
Nitrospinae	0 (0–0)	0 (0–0)	0.30	6	1	3	1
Aquificae	0 (0–0)	0 (0–0)	0.63	2	1	3	2
Armatimonadetes	0 (0–0)	0 (0–0)	0.08	1	0	3	0
Balneolaeota	0 (0–0)	0 (0–0)	0.25	2	2	2	5
Chloroflexi	0 (0–0)	0 (0–0)	0.40	1	1	2	4
Chlorobi	0 (0–0)	0 (0–0)	0.32	1	0	1	0
Chrysiogenetes	0 (0–0)	0 (0–0)	1.00	1	1	1	1
Fibrobacteres	0 (0–0)	0 (0–0)	1.00	1	1	1	1
Nitrospirae	0 (0–0)	0 (0–0)	1.00	1	1	1	1
Deinococcus-Thermus	0 (0–0)	0 (0–0)	1.00	1	1	1	1
Thermodesulfobacteria	0 (0–0)	0 (0–0)	1.00	1	1	1	1
Thermotogae	0 (0–0)	0 (0–0)	1.00	1	1	1	1
Elusimicrobia	0 (0–0)	0 (0–0)	0.15	0	92	0	2
Deferribacteres	0 (0–0)	0 (0–0)	0.32	0	1	0	1

Median (Q1–Q3) abundances of phyla presented as number of reads resulting from 16S rRNA sequencing.

Supplementary Table S3. Abundances of taxa at species level in vegans and omnivores.

Species	Vegans	Omnivores	*p*-Value	Vegans (Max)	Omnivore (Max)	Presence (Vegan)	Presence (Omnivore)
Akkermansia muciniphila	31 (3–667)	37 (1–540.5)	0.426	4197	3591	35	30
Alistipes indistinctus	17.5 (1–76)	27 (4–64.5)	0.257	565	504	27	35
Alistipes onderdonkii	1 (0–12.5)	4 (1–30)	0.177	204	237	22	28
Alistipes putredinis	9 (1.5–33)	22 (2–37)	0.175	114	87	28	32
Allisonella histaminiformans	1 (0–1)	0 (0–1.5)	0.247	46	118	13	8
Alloprevotella rava	1 (0–6)	1 (0–2)	0.619	2044	2941	19	15
Bacteroides coprocola	10.5 (2–43.5)	17 (7–37.5)	0.132	2841	316	23	19
Bacteroides dorei	31 (3–667)	37 (1–540.5)	0.398	314	147	32	34
Bacteroides fragilis	23.5 (7–85.5)	30 (15–85.5)	0.237	2664	396	36	35
Bacteroides massiliensis	4 (2–132.5)	5 (2–165)	0.599	1619	1998	31	30
Bacteroides ovatus	23.5 (7–58)	23 (10–69.5)	0.562	682	344	35	35
Bacteroides plebeius	1 (0–6.5)	0 (0–1.5)	0.121	3245	1063	19	14
Bacteroides uniformis	12.5 (1.5–32.5)	35 (14–71)	**0.004**	150	138	30	33
Bacteroides vulgatus	9.5 (2.5–20)	17.5 (6–34.5)	**0.070**	120	134	31	34
Barnesiella intestinihominis	8.5 (1–27.5)	15 (3.5–30)	0.190	128	187	28	32
Barnesiella viscericola	3 (0.5–6.5)	2 (0.5–5.5)	0.986	101	34	27	27
Bifidobacterium longum	4.5 (1–15.5)	6 (0.5–17)	0.712	299	172	28	27
Butyricicoccus desmolans	5.5 (2–35.5)	2 (1–18)	**0.049**	182	88	33	30
Butyricimonas virosa	0 (0–2)	0.5 (0–5)	0.134	4	26	17	11
Butyrivibrio crossotus	0 (0–1)	0.5 (0–1)	0.248	2335	1118	12	18
Clostridium colinum	3 (0–12)	0 (0–1)	**0.004**	125	73	22	12
Clostridium lactatifermentans	1 (0–3)	2 (0–3.5)	0.321	25	414	23	26
Clostridium leptum	7 (2.5–14.5)	9.5 (5–15)	0.245	259	92	34	35
Collinsella aerofaciens	14 (3.5–31)	22 (4.5–43.5)	0.286	294	722	29	30
Coprobacter fastidiosus	3 (0.5–41)	10.5 (1–34)	0.540	565	504	27	35
Coprococcus eutactus	41.5 (2–226)	31 (0–176.5)	0.366	1173	1676	34	26
Dialister invisus	2 (0–20.5)	4.5 (2–393)	**0.043**	3245	4131	26	34
Dialister succinatiphilus	2.5 (0.5–81.5)	0 (0–2.5)	**0.015**	4047	1831	27	16
Eubacterium coprostanoligenes	57 (18–229.5)	70.5 (30.5–160)	0.982	1870	822	33	36
Eubacterium hallii	6.5 (3–13.5)	9.5 (5.5–14.5)	0.160	82	167	34	36
Faecalibacterium prausnitzii	968.5 (566.5–1774.5)	637 (439.5–1165)	**0.077**	11675	5289	36	36
Finegoldia magna	43.5 (33–63)	44 (31–53)	0.673	136	113	36	36
Gemmiger formicilis	14.5 (5.5–36)	34 (17.5–54.5)	0.038	172	163	35	34
Holdemanella biformis	1 (0–33)	0 (0–38.5)	0.635	484	653	22	17
Leptolyngbya boryana	92 (47–149.5)	85.5 (59.5–141.5)	0.822	320	475	36	36
Megasphaera elsdenii	1.5 (0–5)	1 (0–3.5)	0.496	2240	875	25	23
Odoribacter splanchnicus	48 (19.5–110.5)	46 (18–105)	0.879	205	475	35	33
Parabacteroides distasonis	191 (77–326.5)	175 (87–518.5)	0.669	3176	2999	35	33
Parabacteroides merdae	15.5 (2.5–29.5)	12.5 (1–63)	0.721	260	230	29	28
Paraprevotella clara	1 (0–258.5)	1 (0–123.5)	0.803	1532	2634	20	25
Paraprevotella xylaniphila	0.5 (0–4)	0 (0–1.5)	0.207	108	35	18	13
Parasutterella excrementihominis	6 (1–15)	13 (3.5–96.5)	**0.043**	1798	979	28	33
Phascolarctobacterium faecium	9.5 (1–484.5)	3 (1–401)	0.675	1688	2595	29	28

Species	Vegans	Omnivores	*p*-Value	Vegans (Max)	Omnivore (Max)	Presence (Vegan)	Presence (Omnivore)
Phascolarctobacterium succinatut	1 (0–3)	2 (0–4.5)	0.377	1688	2595	29	28
Planktothrix suspensa	35.5 (18–59)	26.5 (16–77.5)	0.648	273	237	36	36
Prevotella copri	19 (8.5–5250.5)	19 (9–1640.5)	0.765	18976	12158	35	35
Prevotella stercorea	1 (0–6)	1 (0–2.5)	0.393	1876	923	26	25
Ruminococcus bromii	52.5 (2.5–114)	64.5 (22–146.5)	0.477	2004	1277	32	31
Ruminococcus callidus	17.5 (0–35.5)	5.5 (0–27.5)	0.346	83	105	26	24
Sutterella stercoricanis	1 (0–40.5)	0 (0–4.5)	0.106	713	597	23	15
Sutterella wadsworthensis	26.5 (2.5–153)	17 (1–76)	0.312	634	1475	32	28

Median (Q1–Q3) abundances of species presented as number of reads resulting from 16S rRNA sequencing. Only taxa were included that were present in at least 50% of participants with vegan or omnivorous diet and with an absolute abundance of 100 reads in at least one participant. Significant differences with $p \leq 0.05$ were marked in bold.

Supplementary Table S4. Abundances of taxa at genus level in vegans and omnivores.

Genus	Vegans	Omnivores	*p*-Value	Vegan (Max)	Omnivore (Max)	Presence (Vegan)	Presence (Omnivore)
Bacteroides	6352 (1986.2–8377.8)	6094 (3214.8–11222.2)	*0.374*	20765	25858	36	36
Butyricimonas	25.5 (1.8–71.2)	19 (8.5–53.8)	*0.982*	261	2448	28	30
Odoribacter	62 (22.5–115.5)	46.5 (21.5–110)	*0.817*	213	487	35	34
Barnesiella	223 (23.8–826)	327.5 (160–655.2)	*0.424*	3400	3121	34	36
Coprobacter	3 (0.8–38.5)	10.5 (1–32.5)	*0.540*	199	469	27	28
Parabacteroides	454.5 (251.2–698)	550.5 (286.5–1189.8)	*0.295*	3254	5315	36	35
Alloprevotella	1 (0–2)	0.5 (0–2)	*0.801*	2053	2983	20	18
Paraprevotella	22 (0–281.2)	3 (1–135)	*0.510*	1629	2824	26	28
Prevotella	157.5 (27–6527.8)	56.5 (25.8–2219.8)	*0.398*	22873	15903	36	35
Alistipes	638.5 (261.2–1248.8)	752.5 (519.2–1713.2)	*0.311*	3360	4569	36	36
Parasutterella	6 (1–14.5)	13 (3.8–78.8)	***0.045***	1798	982	28	33
Sutterella	76 (22.8–242.2)	49 (3–169.2)	*0.330*	906	2116	34	33
Desulfovibrio	39.5 (16–75.8)	47.5 (14.8–150.8)	*0.401*	648	901	32	34
Haemophilus	2.5 (1–11.5)	0 (0–4)	***0.027***	216	100	28	17
Akkermansia	31 (3–603.5)	37 (1–521.8)	*0.426*	4197	3591	35	30
Bifidobacterium	147 (59.2–343.8)	91 (27.5–341.2)	*0.395*	3254	9975	35	36
Collinsella	22.5 (6.2–45.5)	32 (13.2–55.8)	*0.423*	382	913	31	32
Planktothrix	35.5 (19–59)	26.5 (16–75.8)	*0.648*	273	237	36	36
Leptolyngbya	92 (47.5–147.8)	85.5 (60.8–140.8)	*0.822*	320	475	36	36
Lactobacillus	8 (4–31.8)	5 (2.8–10.2)	*0.051*	106	70	36	34
Streptococcus	8 (5–36.8)	16.5 (6.8–40)	*0.318*	306	185	34	36
Butyricicoccus	114.5 (52.5–209.2)	45.5 (26–92.8)	***0.002***	520	307	36	36
Clostridium	29.5 (18.5–56.8)	22.5 (11.8–43.2)	*0.248*	660	162	36	36
Eubacterium	113 (59–231)	92.5 (63.8–199.2)	*0.848*	1918	863	36	36
Anaerostipes	19 (6.8–34.8)	17 (9–32)	*0.964*	375	413	36	36
Blautia	83.5 (42.5–145)	101.5 (54.8–151.5)	*0.536*	435	362	36	36
Butyrivibrio	1 (0–3)	1.5 (0.8–3)	*0.467*	2338	1118	22	27
Coprococcus	49 (12.8–227.8)	38 (5–181.8)	*0.333*	1177	1696	36	33
Lachnoclostridium	973 (475.8–1259.2)	1091 (445.2–1946.8)	*0.437*	4292	3781	36	36
Roseburia	88.5 (47–219.5)	77.5 (40.2–162)	*0.285*	1314	630	36	36
Tyzzerella	21 (12.5–44)	15 (5.8–25)	*0.107*	127	463	36	34
Oscillibacter	224 (172–377.2)	273 (182.8–358.8)	*0.915*	1073	1251	36	36
Anaerofilum	25 (15–55)	39.5 (22.2–57.8)	*0.338*	185	130	35	36
Faecalibacterium	968.5 (580.8–1764.2)	637 (440.2–1105)	*0.077*	11675	5289	36	36
Gemmiger	14.5 (5.8–35)	34 (18.2–51.8)	*0.038*	172	163	35	34
Ruminiclostridium	113 (39.5–193.8)	96.5 (66.5–126.2)	*0.437*	422	292	36	36
Ruminococcus	245 (124.2–373.2)	325.5 (96.5–614.5)	*0.532*	2567	1741	35	36
Erysipelatoclostridium	13 (7–37)	29.5 (7–44)	*0.327*	166	185	35	35
Holdemanella	1 (0–30.5)	0 (0–38.2)	*0.635*	484	653	22	17
Acidaminococcus	2 (0–7.5)	2.5 (0–6.5)	*0.840*	324	727	22	24
Phascolarctobacterium	72.5 (1.8–524)	21.5 (1–679.5)	*0.955*	1701	2613	33	31
Dialister	392 (6.8–1172.8)	252 (6.8–1283.5)	*0.822*	4591	4640	34	36
Megasphaera	2 (0.8–5.2)	1 (0–4)	*0.275*	2295	919	27	23

Genus	Vegans	Omnivores	p-Value	Vegan (Max)	Omnivore (Max)	Presence (Vegan)	Presence (Omnivore)
Veillonella	8 (4–21.2)	7 (4–13)	*0.491*	169	626	36	35
Finegoldia	43.5 (33.5–63)	44 (31–51.5)	*0.673*	136	113	36	36
Spiroplasma	6.5 (0–30.2)	0 (0–12)	***0.047***	241	95	24	17

Median (Q1–Q3) abundances of genera presented as number of reads resulting from 16S rRNA sequencing. Only taxa were included that were present in at least 50% of participants with vegan or omnivorous diet and with an absolute abundance of 100 reads in at least one participant. Significant differences with $p \leq 0.05$ were marked in bold.
